# Resilience Effects of *SGK1* and *TAP1* DNA Markers during PRRSV Outbreaks in Reproductive Sows

**DOI:** 10.3390/ani10050902

**Published:** 2020-05-22

**Authors:** Marina Laplana, Joan Estany, Lorenzo José Fraile, Ramona Natacha Pena

**Affiliations:** Departament de Ciència Animal, Universitat de Lleida–AGROTECNIO Centre, 25198 Lleida, Spain; marina.laplana@udl.cat (M.L.); joan.estany@udl.cat (J.E.); lorenzo.fraile@udl.cat (L.J.F.)

**Keywords:** PRRSV, sow, pigs, resilience, DNA markers, reproductive traits, mummies

## Abstract

**Simple Summary:**

The genetics of pig resilience is a key issue to select animals with more stable performance when facing unexpected challenges. In pigs, a major stressor is the porcine reproductive and respiratory syndrome virus (PRRSV), which causes serious health problems and productivity drops in farms. In this study, we investigated the role of variants in the *SGK1* and *TAP1* genes using a large dataset of reproductive parameters collected in a farm of Landrace × Large White sows in stable conditions and during a PRRSV outbreak. We showed that all variants affected the reproductive performance in the outbreak, but not during the endemic phase. That is to say, sows carrying certain *SGK1* and *TAP1* genotypes were able to keep up their reproductive performance in spite of the viral outbreak. The number of piglets born alive, stillborn, and mummified piglets were the three parameters more influenced by the genotype of the *SGK1* and *TAP1* markers. Pending validation in other genetic types and farm conditions, these results can have practical applications when planning pig selection and crossbreeding schemes in order to improve resilience to PRRSV.

**Abstract:**

The porcine reproductive and respiratory syndrome virus (PRRSV) is a major infectious stressor that causes serious health problems and productivity drops. Based on previous genome-wide analyses, we selected *SGK1* and *TAP1* as candidate genes for resilience, and genotyped three mutations, including a 3′UTR variant SGK1_rs338508371 and two synonymous variants TAP1_rs1109026889 and TAP1_rs80928141 in 305 Landrace × Large White sows. All polymorphisms affected the reproductive performance in the outbreak, but not during the endemic phase, thereby indicating a potential use of these markers for resilience. Moreover, some genotypes were associated with a stable performance across PRRSV phases. Thus, in the outbreak, the SGK1_rs338508371 AA sows had less piglets born alive (*p* < 0.0001) and more stillborns (*p* < 0.05) while other sows were able to keep their productivity. During the outbreak, TAP1_rs80928141 GG sows had less piglets born alive (*p* < 0.05) and both TAP1 polymorphisms influenced the number of mummies in an additive manner (*p* < 0.05). Remarkably, TAP1_rs80928141 AA sows had around one mummy more than GG sows (*p* < 0.01). Resilience to PRRSV could be improved by including the *SGK1* and *TAP1* markers in crossbreeding and/or selection schemes, as they contribute to maintaining a stable number of piglets born alive and lost, particularly mummies, despite the outbreak.

## 1. Introduction

Every day, pig farms face a number of stressors that can modify the pig’s normal physiology and thus alter their productivity. Among the potential stressors that can challenge a farm, immunological stressors (i.e., infectious agents) can have dramatic consequences leading to large losses in pig farms. The selection of pigs with an inherent capacity to cope with such stressors has been proposed as a sustainable strategy that can complement advances in health management of the farms [1].

The porcine reproductive and respiratory syndrome virus (PRRSV) is a major immunological challenge which can represent a yearly cost of almost 126 €/sow in Europe and a total of $ 650M/year in the United States [2,3,4]. There are a wide variety of clinical manifestations of PRRSV between herds, which depend on the virulence of the strain and on the age and previous immune responses of the pig [5]. In sows, PRRSV infection can lead to reproductive failure, including embryonic death in early gestation [6,7], foetal death, and abortions in late gestation, early farrowings, and elevated preweaning mortality [8,9]. Although PRRSV vaccines are available and have been improved over the last two decades, the high mutation rate of the virus and its ability to “confuse” the innate immune responses compromises the capacity of vaccines on controlling this disease [10]. Thus, there is a need to develop new tools that allow the selection of resilient animals that help to mitigate the current situation and reduce the impact of PRRSV outbreaks.

The genetic selection of pigs has been very successful at improving a number of traits, including longevity, production, and meat quality parameters, as well as maternal traits [11]. Several studies have demonstrated the role of genetic variability of the host in the outcome of PRRSV infection both for the respiratory form in growing pigs [12,13,14,15,16,17,18] and in pregnant sows [19,20,21]. Altogether, the data compiled by these studies indicate the potential use of genetic selection to improve sow’s resilience in reproductive traits under the challenge of a PRRSV outbreak. 

In this line, our project aims to contribute to the genetic selection of resilient pigs by finding genetic markers associated with stable reproductive performance during a PRRSV infection. Among previously reported regions influencing reproductive performance (number of stillborn) and immunity response (antibody levels) in sows during a PRRSV outbreak [21], we decided to focus on two quantitative trait loci (QTL) regions located in chromosomes (SSC) 1 and 7 related to immune responses. We found two promising candidate genes: serum and glucocorticoid-regulated kinase 1 (*SGK1)* and transporter associated with antigen processing-1 (*TAP1)*. SGK1 has been implicated in immune homeostasis and tolerance, inflammation, and cell survival during early embryogenesis [22,23]. On the other hand, TAP1 is an adenosine triphosphate (ATP)-binding cassette subfamily B transporter mostly expressed in blood and immune cells. It is involved in the pumping of peptides from reticulum to membrane, where they are presented by class I major histocompatibility complex (MHC) molecules. The expression of *TAP1* is elevated by several viruses, including PRRSV [24,25]. Both candidates are related to host immune responses and might contribute to keeping a health balance for successful pregnancy development. Thus, we screened *SGK1* and *TAP1* for genetic variability to be validated as genetic markers for resilience to PRRSV infection. 

## 2. Materials and Methods 

### 2.1. Animals

A farm of Landrace × Large White sows from a large integration Spanish company (Pinsos del Segre S.A, Lleida, Spain) was included in the study. The main characteristics of the animals and farm have been previously described [19]. Shortly, four batches of six or seven-week-old female piglets were vaccinated with a PRRSV-modified live vaccine following the manufacturer’s recommendations (2 mL by intramuscular dose that is equivalent to 10^5^ TCID_50_ of PRRSV DV strain by animal) [19].

Pigs were transferred to a PRRSV-positive stable production farm [26], following the standard operation procedures in course at the company. In the present study, 305 sows were followed for a period of almost three years, from 2016 to 2018, delivering 1464 farrowings. No major pig diseases were reported during the follow-up, with the exception of a PRRSV outbreak at the end of the screened period, which lasted 15 weeks. All sows that underwent the outbreak had previous data during the endemic situation. The epidemic status of the farm was confirmed by standard laboratorial procedures as follows [19,27]. PRRSV exposure was confirmed in all sows by determining antibodies against this virus at the PRRSV outbreak. As animals were not vaccinated at the production farm, the presence of antibodies against the virus implies exposure to a PRRSV field strain. PRRSV antibody titre was determined (sample-to-positive ratio) by ELISA (IDEXX PRRS X3, IDEXX laboratories Inc, Westbrook, ME, USA) as previously described [19]. The prevalence of PRRSV by antibody detection was 100% in the included sows. Data on 1464 farrowings were recorded including the farrowing date and the number of piglets born alive (NBA), stillborn (NSB), and mummified (NMU) per litter. The total number of lost piglets per litter (NLP) was calculated as the sum of NSB and NMU and the total number of piglets born per litter (TNB) as the sum of NBA and NLP. The proportion of lost piglets (%LP) was expressed as the percentage of NLP over TNB. The description of the litter size data used in this study has been previously described in [19]. All experimental procedures were approved by the Ethics Committee for Animal Experimentation of the University of Lleida and performed in accordance with authorisation 7700 issued by the Catalan Department of Agriculture, Livestock, Fisheries and Food (Section of biodiversity and hunting). 

### 2.2. DNA Samples

Genomic DNA from 305 sows was extracted from peripheral blood mononuclear cells (PBMCs) by standard protocols as described in [28]. First, PBMCs were incubated with proteinase K and lysis buffer and then treated with RNaseI prior to be subjected to phenol/chloroform purification and isopropanol precipitation as in [28]. DNA concentration and purity were assessed by Nanodrop-100 (Thermo Fisher Scientific, Waltham, MA, USA).

### 2.3. cDNA Samples

Total RNA was isolated from the tonsils of 16 Landrace × Large White pigs (4.5 month-old) available in the laboratory from other pigs of the same genetic background following the indication of TRI-reagent (Sigma Aldrich, Tres Cantos, Spain). Two micrograms of RNA were first treated with ezDNase (Invitrogen, Carlsbad, CA, USA) and retrotranscribed into cDNA using the SuperScript IV reverse transcriptase (Invitrogen, Carlsbad, CA, USA) with a combination of oligo (dT) and random hexamer primers following the supplier’s protocol. cDNA samples were used to screen for variation in small exons flanked by large introns as described in the following section.

### 2.4. Characterisation of SGK1 and TAP1 Genetic Variability

The genetic structure of selected genes was analysed in Sscrofa11.1 genome assembly using Ensembl website (www.ensembl.org). The genomic sequence of selected candidate loci was retrieved from Ensembl and used to design primer pairs for PCR amplification with Primer3Plus software (www.bioinformatics.nl/cgi-bin/primer3plus/primer3plus.cgi). The *SGK1* gene was amplified in eight PCR fragments and *TAP1* in four fragments. The complete list of PCR primers used is shown in Supplementary file 1, Table S1. Gene fragments were amplified in 16 sows (eight with stable performance across nonoutbreak and outbreak situations, and eight with severe reproductive failure during the outbreak). PCR reactions were performed in a final volume of 25 μL including 1× NH_4_ reaction buffer, 2 mM MgCl_2_, 0.16 mM dNTPs, 0.4 μM each primer, 1 U of Biotaq DNA polymerase (Bioline, London, UK) and 60 ng of DNA with the following cycling parameters: 95 °C 5 min and 35 cycles of 95 °C 15 sec, 60 °C 30 sec and 72 °C 1 min with a final extension step at 72 °C for 7 min using an Applied Biosystems^®^ Veriti^®^ 96-Well Thermal Cycler (Applied Biosystems, Thermo Fisher Scientific, Waltham, MA, USA). When tonsil cDNA was used as template, 1 μL of cDNA was used as PCR input. All amplification products were run in agarose gels and correct size and specificity of the products was evaluated under UV visualisation. PCR products were purified with NZY GelPure (NZYtech, Lisboa, Portugal) following the manufacturer’s recommendations and sequenced with an ABI 3730 xL sequencer (Stabvida Lda., Caparica, Portugal). Finally, ChromasPro v2.1.8 (Technelysium Pty Ltd., South Brisbane, Australia) was used for sequence alignment and comparison to screen for genetic variability.

### 2.5. Genotyping of Selected Mutations

**Figure 1:**
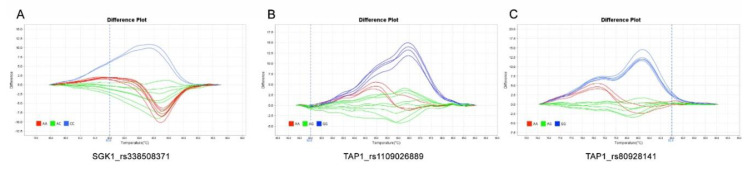
High-resolution melting (HRM) standardised genotyping images of the three tested polymorphisms, SGK1_rs338508371 (**a**), TAP1_rs1109026889 (**b**), and TAP1_rs80928141 (**c**) genotypes.

Three polymorphic positions were selected for further analysis, including a 3′UTR variant of *SGK1* gene, SGK1_rs338508371, C > A; and two synonymous variants in *TAP1* gene, TAP1_rs1109026889, G > A in exon 1 and TAP1_rs80928141, G > A in exon 5 (Table 1). Genotyping was performed by PCR amplification followed by high-resolution melting (HRM) analysis (Figure 1). Primers for HRM genotyping were design using Primer3Plus software with preestablished qPCR settings and limiting the product size to 60–120 bp (Supplementary file 1, Table S2). Selected variants were genotyped in the 305 Landrace × Large White sows by PCR amplification in a QuantStudio 3 v1.4 thermocycler with QuantStudio Design & Analysis Software (Applied Biosystems, Thermo Fisher Scientific, Waltham, MA, USA). The PCR reaction was performed in a final volume of 6 μL including 1× Thermo Scientific™ Luminaris Color HRM qPCR Master Mix (Thermo Fisher Scientific, Waltham, MA, USA), 0.3 μM of each primer, and 10 ng of genomic DNA with the following cycling parameters: 50 °C 2 min, 95 °C 10 min, and 40 cycles of 95 °C 15 sec, 60 °C 1 min, followed by a high-resolution melting curve starting with a denaturation at 95 °C for 15 sec, annealing at 60 °C for 1 min and a slow ramp at 0.015 °C/sec up to 95 °C. High Resolution Melt software v3.1 (Applied Biosystems, Thermo Fisher Scientific, Waltham, MA, USA) was used for the melting data analysis and the genotyping of the samples.

Linkage Disequilibrium (LD) analysis between markers was performed using Haploview 4.2 [29]. 

### 2.6. Statistical Analysis

The effect of the three DNA markers on reproductive traits has been evaluated using a mixed model which included the batch (four levels), the parity cycle (three levels; 1, 2, and 3 or more), the marker genotype (three genotypes), the PRRSV health status (endemic or outbreak), and the interaction between marker genotype and PRRSV health status as fixed factors, and the sow as a random factor. In addition, TNB was included as a covariate when analysing NSB, NMU, and NLP. The effect of each genetic marker was tested following an F-test and multiple pairwise comparisons among genotypes were done using the Tukey test. *P*-values were corrected for multiple comparisons using the Benjamini–Hochberg False Discovery Rate P-Value adjustment. All analyses were performed with the JMP Pro14 (SAS Institute Inc, Cary, NC, USA) software.

## 3. Results

### 3.1. SGK1 and TAP1 Genetic Variability

We screened *SGK1* and *TAP1* loci for genetic variability covering all coding exons, and 5′/3′UTRs of both genes. The *SGK1* gene expands 127.83 kb and codes for four protein coding transcripts. *SGK1* transcript ENSSSCT00000062973.2 was not detectable in cDNA samples, thus, only shorter transcripts containing 13 exons were sequenced. *TAP1* expands 9.09 kb and contains 12 exons that code for two protein coding transcripts with the shorter transcript lacking exon 7.

We identified a total of 16 variable sites, eight in *SGK1* gene (two variants in 5′UTR, one synonymous SNP in exon 17, and five in 3′UTR) and eight in *TAP1* (all synonymous) (Supplementary file 1, Table S3). From all reported mutations, we selected three variants to be further analysed based on the feasibility to establish a HRM qPCR protocol and the segregating frequency: SGK1_rs338508371, with alleles C/A, located at the common 3′UTR of all *SGK1* transcripts and two synonymous mutations for *TAP1* with alleles G/A, TAP1_rs1109026889 and TAP1_rs80928141, located in the common first and fifth exons, respectively. Genotyping analysis of the selected variants in the reproductive dataset reported a minor allele frequency (MAF) of 0.49 for SGK1_rs338508371 A allele, 0.33 for TAP1_rs1109026889 G allele and 0.35 for TAP1_rs80928141 A allele. The genotype distribution of candidate variants is shown in Table 2. LD analysis showed no relevant linkage disequilibrium between *TAP1* markers (D’ = 0.07) or between *SGK1* and *TAP1* markers (D’ < 0.17).

### 3.2. Association of SGK1 and TAP1 Markers with Reproductive Traits and Resilience

As expected, the outbreak resulted in a general drop in the reproductive performance of the farm, with less NBA and more NLP per litter (see [19] for more details). In this study, we have used the reproductive data as a way to identify resilience in sows. Thus, if sows are able to keep NBA and NLP stable despite the outbreak, this is indicative of a better ability to cope with the challenge of a PRRSV infection. 

**Figure 2:**
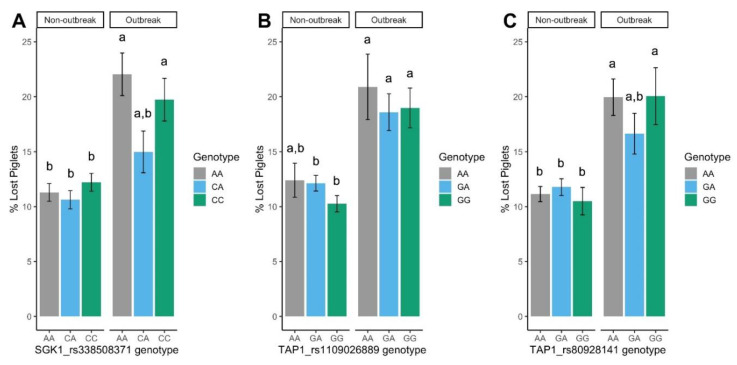
Least square means by PRRSV health status for the percentage of lost piglets per litter by SGK1_rs338508371 (**a**), TAP1_rs1109026889 (**b**), and TAP1_rs80928141 (**c**) genotypes. Error bars represent standard errors. Within markers, means with different letters differ significantly (*p* < 0.05).

In this study, we detected a significant interaction between the SGK1_rs338508371 marker and the epidemic status for NBA, NMU, NLP (*p* < 0.05; Table 3) and %LP (*p* < 0.05, Figure 2). Sows’ performance did not differ between SGK1_rs338508371 genotypes while the farm was in nonoutbreak situation. Differences were only evident during the PRRSV outbreak, indicating a putative role of SGK1_rs338508371 marker in resilience. During the outbreak, AA sows had around two fewer piglets born alive per parity than CA sows (9.9 piglets vs. 12.1 piglets, respectively, *p* < 0.01). This difference was due to a drop in the NBA in AA sows between nonoutbreak and outbreak phases of the disease, which did not happen in CA or CC sows (*p* < 0.01; Table 3). In the same line, the NLP was higher in sows with AA genotype due to a rise in the NSB and NMU (*p* < 0.05). Similarly, the %LP nearly doubled in litters from AA sows during the PRRSV outbreak compared with the endemic situation (22.0% vs. 11.3%, respectively) and increased by around 50% in CC sows (19.8% vs. 12.2 %, respectively) (Figure 2, *p* < 0.01). In contrast, the change in the percentage of lost piglets was subtler and not significant in litters from CA sows, indicating more resilience overall when faced with a PRRSV infection than the other genotypes.

Furthermore, we found significant results for reproductive traits for both *TAP1* markers. We found an interaction between the marker and the epidemic status of the farm in the NMU for both *TAP1* markers (*p* < 0.05; Table 4 and Table 5). As for SGK1_rs338508371, the performance of the sows did not differ between *TAP1* genotypes when the farm was under the endemic phase (except for TNB, in the rs1109026889 marker). In contrast, during a PRRSV outbreak, the TAP1_rs1109026889 GA sows had around 0.40 NMU less per parity (*p* < 0.05) as compared to GG sows (0.76 piglets vs. 1.16 piglets, respectively). Similar results were found for TAP1_rs80928141 GG sows, which had one less mummy per farrowing than AA sows (0.31 vs. 1.31 piglets, *p* < 0.01), with heterozygote animals showing intermediate values (*p* < 0.05). The two *TAP1* markers displayed an additive behaviour over the NMU, with an allele substitution effect of A for G of −0.50 ± 0.07 mummies (rs80928141, *p* < 0.01) and +0.19 ± 0.09 mummies (rs1109026889, *p* < 0.05). In this context, TAP1_rs1109026889 GA and TAP1_rs80928141 GG sows are able to reduce the impact of PRRSV infection in the reproductive performance through a lower raise in NMU as compared to the other genotypes. In the outbreak situation, TAP1_rs80928141 also behaved additively over the NSB (substitution effect of A for G of +0.42 ± 0.19, *p* < 0.05), which explains the drop in nearly two piglets born alive in GG sows between endemic and outbreak situations (*p* < 0.05). 

## 4. Discussion

The current trends of global population growth rates highlight the necessity of improving the mechanisms of food production, not only by increasing the efficiency of the production itself, but also by developing more sustainable strategies. In this line, several approaches have been implemented over the last 40 years in order to select animals for production traits. A number of studies have reported that highly selected livestock are more at risk for behavioural, physiological, and immunological problems and respond worse to stressors [30,31,32]. 

Among the potential stressors that may affect production and reproductive parameters in pigs, the porcine reproductive and respiratory syndrome virus (PRRSV) stands out as one of the most economically important diseases. PRRSV infection reduces pig growth in production farms and alters reproductive parameters by increasing the number of abortions and the number of stillborn and mummified piglets per farrowing. A genome-wide association study using data from the PRRS Host Genetics Consortium PRRS-CAP project found a QTL region in chromosome 4 that explained 15.7% and 11.2% of the genetic variability for viral load and weight gain in growing pigs [14], but was not associated with better reproductive performance during PRRSV outbreaks in sows [21]. Fewer studies have addressed the impact of genetic variability in the resilience of sows faced with a PRRSV challenge. Serao et al. [21] showed how heritability of the different traits varies during a pre-PRRSV phase and a PRRSV infection phase. The NBA had the most stable heritability across these phases with estimates of 0.08 and 0.09, respectively. On the other hand, NSB and NMU showed broader changes in the heritability estimates across PRRSV phases. All this indicates a different behaviour of reproductive traits under the effect of an external stressor, such as PRRSV infection. Moreover, they reported a list of regions that accumulated more than 0.5% of the total genetic variance explained by the markers for NSB and antibody levels during the PRRSV phase. 

In this study, we evaluated two genes located within the genomic windows described by Serao and coworkers [21]. These are *SGK1* and *TAP1*, in SSC1 (29.7 Mb) and SSC7 (25.0 Mb), respectively. We selected three variable positions in these genes to be tested for association with reproductive traits under PRRSV endemic and PRRSV outbreak situations. In a positive-stable farm (endemic situation), a small amount of virus recircularisation, leading to no major drops in piglets born alive, is expected [26]. With the exception of TAP1_rs1109026889 for TNB, the markers were not associated with differences in the reproductive performance during the endemic phase. However, for the three markers, differences between genotypes emerged at the outbreak phase, thereby reflecting their putative function during the infection process and their relevance in determining the resilience of the animal. In all cases, the effects described for each individual maker and their interaction with the health status of the farm did not change when all the markers were fitted into the same model. 

Thus, during the outbreak, sows with the AA genotypes for SGK1_rs338508371 had about two less piglets born alive than during the endemic phase. This drop in NBA did not take place in the other two genotypes, which were able to perform at the same level in both phases of the disease. The AA sows also had more mummies per litter, which resulted in more losses (NLP). These results match with the effect previously described in this genomic region of SSC1 associated with lost piglets during a PRRSV infection [21]. Thus, SGK1_rs338508371 AA sows show worse reproductive performance during PRRSV outbreak, which could be attributed to a lower capacity to overcome this challenge. According to this, breeding strategies leading to avoid the AA genotype in crossbred sows would promote a more resilient phenotype when faced with a PRRSV infection. 

The mechanisms by which this *SGK1* marker can improve a sow’s performance during the outbreak are still unknown. *SGK1* was initially identified as an immediate–early gene stimulated transcriptionally by serum and glucocorticoids [33]. In mice, SGK1 is implicated with cell survival during early embryogenesis [22] and its deregulation in the endometrium can cause reproductive failure by interfering with embryo implantation or predisposing to pregnancy complications [33,34,35]. SGK1_rs338508371 is located at the 3′UTR of the gene with a possible implication in mRNA stability and protein synthesis. No miRNA binding sites have been reported overlapping the polymorphism position; however, a functional effect of the variant cannot be ruled out. In addition, a direct role of *SGK1* during infections has not yet been reported.

Regarding *TAP1*, in this study, we tested two synonymous variants, TAP1_rs1109026889 and TAP1_rs80928141, located in the first and fifth exon of the gene, respectively. Despite their close proximity (~3 kb), the two markers did not show a marked linked disequilibrium and probably neither of them are causal mutations. Both variants impacted the NMU during the epidemic phase of the disease, but not during the endemic phase. During the PRRSV outbreak, TAP1_rs1109026889 GA and TAP1_rs80928141 GG sows had around 0.4 and 1 NMU less per parity as compared to GG and AA sows, respectively. These effects behaved additively, and therefore would respond to selection. However, TAP1_rs80928141 has an additive effect over NSB in the opposite direction that NMU, balancing the final number or losses over the three genotypes. 

*TAP1* is mostly expressed in blood and immune cells in pigs and has been associated with immune response through the major histocompatibility complex (MHC) class I-mediated antigenic presentation [24]. MHCI molecules play an important role mediating immune tolerance at the maternal–foetal interface and interacting with the innate and the adaptive immune systems in the context of preimplantation and early pregnancy. In addition, this gene is upregulated after infecting pig white blood cells in vitro with PRRSV [24], which indicates a role in activating the MHCI pathway after PRRSV infection. The studied polymorphisms do not involve a change in the amino acid sequence of the protein; however, they may have an indirect effect on the protein function through alteration of gene expression. These variants may alter regulatory elements such as enhancers, chromatin interactions, or transcription factor binding sites, which can have an effect on the expression of *TAP1* or related genes. Further functional characterisation of the variants will be required to unravel their role in resilience.

Taken together, all this information illustrates the opportunity of improving resilience against PRRSV by including these markers in crossbreeding and/or selection schemes. The CA and CC sows for the SGK1_rs338508371 and GA and AA sows for TAP1_rs80928141 can keep up a stable NBA despite the PRRSV epidemic. This is particularly relevant for the overall productivity of the farm. In addition to maintaining reproductive performance under PRRSV outbreaks, the implementation of resilience markers would contribute to increasing animal welfare and farm productivity, while reducing economic losses and the use of veterinary drugs. Before implementation, results need to be validated in sows of other genetic types and with more data in order to rule out any spurious associations. Our group has recently published results on other DNA markers related to lower abortion rates during PRRSV infections [20] that could be added to those described here to form a potential panel to be validated in future studies with other populations.

## 5. Conclusions

Our results indicate that genetic variation at *SGK1* and *TAP1* are potential markers for resilience in PRRSV-infected sows as they are associated with stable reproductive outcomes during PRRSV outbreaks without exhibiting negative effects on the sow’s performance during endemic situations.

## Figures and Tables

**Table 1:** Description of *SGK1* and *TAP1* polymorphisms and allele frequency of the minor allele.

Locus	Marker	Genomic Location (Sscrofa11.1)	Alleles	Gene Region	Protein Effect	MAF^1^ (allele)
*SGK1*	rs338508371	1:29753070	C/A	3’UTR	noncoding	0.49 (A)
*TAP1*	rs1109026889	7:25071346	G/A	exon 1	synonymous	0.34 (A)
*TAP1*	rs80928141	7:25068055	G/A	exon 5	synonymous	0.33 (G)

^1^ Minor allele frequency (MAF).

**Table 2:** Number of sows and farrowings per *SGK1* and *TAP1* genotypes and porcine reproductive and respiratory syndrome virus (PRRSV) health status of the farm.

PRRSV Health Status	Descriptors	SGK1_rs338508371			TAP1_rs1109026889			TAP1_rs80928141		
		AA	CA	CC	AA	GA	GG	AA	GA	GG
Nonoutbreak	sows	95	97	97	31	134	118	139	111	41
	farrowings	352	332	402	94	485	481	543	401	146
Outbreak	sows	55	59	55	23	79	65	78	62	30
	farrowings	55	59	55	23	79	65	78	62	30

**Table 3:** Least square means (± SE) for reproductive traits by SGK1_rs338508371 genotype and farm health status. Significant interactions between genotype and the health status of the farm (SGK1*Status) are indicated.

n. Farrowings	Nonoutbreak			Outbreak			SGK1*Status	
	AA	CA	CC	AA	CA	CC		
	352	332	402	55	59	55	*p*-Value	FDR
TNB	13.12 ± 0.26	13.81 ± 0.26	13.92 ± 0.26	13.12 ± 0.49	14.59 ± 0.48	14.50 ± 0.49	n.s.	n.s.
NBA	11.55 ± 0.22 ^a^	12.20 ± 0.22 ^a^	12.08 ± 0.22 ^a^	9.91 ± 0.45 ^b^	12.10 ± 0.44 ^a^	11.37 ± 0.45 ^a,b^	<0.01	0.02
NSB	1.71 ± 0.11 ^b^	1.55 ± 0.11 ^b^	1.70 ± 0.11 ^b^	2.29 ± 0.26 ^a^	1.56 ± 0.25 ^b^	1.88 ± 0.26 ^a,b^	n.s.	n.s.
NMU	0.11 ± 0.04 ^c^	0.09 ± 0.04 ^c^	0.11 ± 0.04 ^c^	1.12 ± 0.10 ^a^	0.70 ± 0.10 ^b^	1.01 ± 0.10 ^a,b^	0.02	0.03
NLP	1.82 ± 0.12 ^c^	1.64 ± 0.12 ^c^	1.81 ± 0.12 ^c^	3.42 ± 0.28 ^a^	2.27 ± 0.27 ^b,c^	2.90 ± 0.28 ^a,b^	0.03	0.06

TNB—total number of piglets born per farrowing; NBA—number of piglets born alive; NSB—number of stillborns; NMU—number of mummies; NLP—number of lost piglets (NSB + NMU); FDR—false discovery rate; n.s. —not significant. Within trait, means not connected by the same letter indicate significant differences (*p* < 0.05).

**Table 4:** Least square means (± SE) for reproductive traits by TAP1_rs1109026889 genotype and farm health status. Significant interactions between genotype and the health status of the farm (TAP1*Status) are indicated.

n. Farrowings	Nonoutbreak			Outbreak			TAP1*Status	
	AA	GA	GG	AA	GA	GG		
	94	485	481	23	79	65	*p*-Value	FDR
TNB	13.37 ± 0.47 ^a,b^	14.09 ± 0.21 ^a^	13.10 ± 0.23 ^b^	13.39 ± 0.75 ^a,b^	14.75 ± 0.41 ^a^	13.48 ± 0.45 ^a,b^	n.s.	n.s.
NBA	12.02 ± 0.22 ^a,b^	12.05 ± 0.10 ª	12.23 ± 0.11 ^a,b^	10.64 ± 0.42 ^a,b^	11.09 ± 0.24 ^a,b^	11.00 ± 0.26 ^b^	n.s.	n.s.
NSB	1.69 ± 0.21	1.73 ± 0.10	1.54 ± 0.10	2.29 ± 0.40	2.02 ± 0.22	1.71 ± 0.24	n.s.	n.s.
NMU	0.17 ± 0.08 ^c^	0.08 ± 0.04 ^c^	0.11 ± 0.04 ^c^	0.93 ± 0.16 ^a,b^	0.76 ± 0.09 ^b^	1.16 ± 0.10 ^a^	0.02	0.03
NLP	1.85 ± 0.22 ^a^	1.82 ± 0.10 ^a^	1.64 ± 0.11 ^a^	3.23 ± 0.42 ^b^	2.79 ± 0.24 ^b^	2.88 ± 0.26 ^b^	n.s.	n.s.

TNB—total number of piglets born per farrowing; NBA—number of piglets born alive; NSB—number of stillborns; NMU—number of mummies; NLP—number of lost piglets (NSB+NMU); FDR—false discovery rate; n.s. —not significant. Within trait, means not connected by the same letter indicate significant differences (*p* < 0.05).

**Table 5:** Least square means (± SE) for reproductive traits by TAP1_rs80928141 genotype and farm health status. Significant interactions between genotype and the health status of the farm (TAP1*Status) are indicated.

n. Farrowings	Nonoutbreak			Outbreak			TAP1*Status	
	AA	GA	GG	AA	GA	GG		
	543	401	146	78	62	30	*p*-Value	FDR
TNB	13.54 ± 0.22	13.50 ± 0.24	13.90 ± 0.40	14.61 ± 0.42	13.80 ± 0.47	13.25 ± 0.67	n.s.	n.s.
NBA	11.88 ± 0.18 ^a^^,b^	11.78 ± 0.21 ^a,b^	12.34 ± 0.34 ª	11.44 ± 0.38 ^a,b^	11.09 ± 0.43 ^a,b^	10.55 ± 0.61 ^b^	n.s.	n.s.
NSB	1.66 ± 0.09 ^a,b^	1.70 ± 0.10 ^a,b^	1.48 ± 0.17 ^b^	1.64 ± 0.22 ^a,b^	1.89 ± 0.25 ^a^	2.56 ± 0.34 ^a^	0.03	0.05
NMU	0.10 ± 0.03 ^c^	0.11 ± 0.04 ^c^	0.09 ± 0.06 ^c^	1.31 ± 0.09 ^a^	0.78 ± 0.10 ^b^	0.31 ± 0.14 ^c^	<0.01	<0.01
NLP	1.76 ± 0.10 ^c^	1.82 ± 0.11 ^b,c^	1.57 ± 0.18 ^c^	2.95 ± 0.24 ^a^	2.68 ± 0.26 ^a^	2.87 ± 0.37 ^a,b^	n.s.	n.s.

TNB—total number of piglets born per farrowing; NBA—number of piglets born alive; NSB—number of stillborns; NMU—number of mummies; NLP—number of lost piglets (NSB+NMU); FDR—false discovery rate; n.s. —not significant. Within trait, means not connected by the same letter indicate significant differences (*p* < 0.05).

## Supplementary Materials

The following are available online at https://www.mdpi.com/2076-2615/10/5/902/s1, Table S1: description of primers used for the amplification of the *SGK1* and *TAP1* genes, Table S2: primers used in the high resolution melting genotyping protocols, Table S3: list of polymorphisms identified in the regulatory and coding sequences of the *SGK1* and *TAP1* genes.
