# Supplementary material for: Resilience Effects of SGK1 and TAP1 DNA Markers during PRRSV Outbreaks in Reproductive Sows
Source: Animals (Basel). 2020 May 22;10(5):902. doi: 10.3390/ani10050902 (PMC7278433; doi:10.3390/ani10050902)
Supplement: Supplementary file 1 [file animals-10-00902-s001.pdf]

animals

MDPI

Resilience Effects of *SGK1* and *TAP1* DNA Markers During PRRSV Outbreaks in Reproductive Sows

Marina Laplana, Joan Estany, Lorenzo José Fraile and Ramona Natacha Pena *

Departament de Ciència Animal, Universitat de Lleida–AGROTECNIO Centre, 25198 Lleida, Spain; marina.laplana@udl.cat (M.L.); joan.estany@udl.cat (J.E.); lorenzo.fraile@udl.cat (L.J.F.)

* Correspondence: romi.pena@udl.cat; Tel.: +34-973-702-918

Table S1. Description of primers used for the amplification of the *SGK1* and *TAP1* genes.

Locus	Region Name	Primer Sequences (5'→ 3') Forward	Reverse	PCR size (bp)	Transcript ID [1]	PCR template
SGK1	SGK1_exon1	TCTCCCTCTGCTTTTCTGGC	AACCTGTCCTTACCCGCTTC	629	ENSSSCT00000062973.2	Genomic
	SGK1_exon2	CTTTGCAGCTTGCTCCCTTC	CCACCCGTCCTCTGAAACAT	320	ENSSSCT00000037228.1	Genomic
	SGK1_exon2	GGAGGCACCGGCTAATCTTT	ACTCCTTTTCTGCACCTCGG	815	ENSSSCT00000037646.1	Genomic
	SGK1_exon1	GCCACTTCCTCATTGTTGCG	CAACGCCACGCACACTAATC	290	ENSSSCT00000027054.3	Genomic
	SGK1_exon13	CCTGGACCTCTTGACCCTGA	AAACACCAGGCTCTGACTGA	980	ENSSSCT00000037228.1	Genomic
	SGK1_exon1-9	CTGTGAACTCCCCACTGTGG	GTAACCCAAGGCACTGGCTA	953	ENSSSCT00000062973.2	cDNA
	SGK1_exon3-9	TGAAACAGAGAAGGATGGGC	TGTGTCCCTGTGAATCTAGCA	513	ENSSSCT00000037228.1	cDNA
	SGK1_exon7-13	CGGTGGAGAGTTGTTCTACCA	TCGGCTGCTTCCTTGACG	708	ENSSSCT00000037228.1	cDNA
TAP1	TAP1_exon1	TCGTTTTCCCTTTCCCATAA	GTCAGAAGGAGCCAGAGGTG	778	ENSSSCT00000001630.4	Genomic
	TAP1_exon 11	GTGGAGTCGCTCCTGTATGAA	AAATACCAAGAATCCACAGGGTA	603	ENSSSCT00000001630.4	Genomic
	TAP1_exon1-7	CTCTGGCTCCTTCTGACCCT	AGGTGGACAGCAGTACCTGA	830	ENSSSCT00000001630.4	cDNA
	TAP1_exon6-11	AGCGGAAACCTCGTCACATT	AGGATGTGATTGGCCTGCTC	818	ENSSSCT00000001630.4	cDNA

[1] Based on Ensembl release 99, Sscrofa11.1 (GCA_000003025.6).

Table S2. Primers used in the high-resolution melting genotyping protocols.

Locus	Target Polymorphism	Forward Primer Sequence (5'→3')	Reverse Primer Sequence (5'→ 3')	Amplicon Size (bp)
SGK1	rs338508371	TGTTGCTCCTCGTGGACTTC	GTGTCCTGCAAGTGTCACATTG	101
TAP1	rs1109026889	CTTCGCCCTCAGCTATGC	TGACTTCCTTGCACCCAAAG	81
TAP1	rs80928141	CGGAGCTTTGCCAATGAAGAG	TGAGTGTCTTCATGTCCTGCAG	70

Table S3. List of polymorphisms identified in the regulatory and coding sequences of the *SGK1* and *TAP1* genes.

Locus	Location Within Transcript [1]	Mutation Type	Gene Location	Gene Effect	Genomic Location (SScrofa11.1)	rs number
SGK1	c.-726T>C	SNP	Exon 1	5′UTR	1:29626049	rs31849774
	c.-708A>G	SNP	Exon 1	5′UTR	1:29626067	rs329854265
	c.1122T>C	SNP	Exon 17	Synonymous	1:29752467	rs332443855
	c.*8A>T	SNP	Exon 17	3′UTR	1:29752568	rs345836515
	c.*269indelG	indel	Exon 17	3′UTR	1:29752829	
	c.*350A>G	SNP	Exon 17	3′UTR	1:29752910	rs337827000
	c.*433G>A	SNP	Exon 17	3′UTR	1:29752993	rs332161325
	**c.*510C>A**	**SNP**	**Exon 17**	**3′UTR**	**1:29753070**	**rs338508371**
TAP1	**c.+517G>A**	**SNP**	**Exon 1**	**Synonymous GCG/GCA A141**	**7:25071346**	**rs1109026889**
	c.+729G>A	SNP	Exon 3	Synonymous GGG/GGA G243	7:25070440	
	c.+942G>A	SNP	Exon 4	Synonymous GGG/GGA G314	7:25068693	rs1114053665
	**c.+1155G>A**	**SNP**	**Exon 5**	**Synonymous AAG/AAA K385**	**7:25068055**	**rs80928141**
	c.+1608C>G	SNP	Exon 9	Synonymous CCC/CCG P536	7:25065461	
	c.+1707T>C	SNP	Exon 10	Synonymous TAT/TAC Y569	7:25065138	
	c.+1749C>T	SNP	Exon 10	Synonymous GCC/GCT A583	7:25065096	rs1113696702
	c.+1900C>T	SNP	Exon 11	Synonymous CTG/TTC L634	7:25064167	rs80955756

[1] Transcript used for polymorphism location: *SGK1*-ENSSSCT00000062973; *TAP1*-ENSSSCT00000001630.4; Polymorphisms genotyped in this study are highlighted in bold.
